# Stevioside reduces inflammation in periodontitis by changing the oral bacterial composition and inhibiting *P. gingivalis* in mice

**DOI:** 10.1186/s12903-023-03229-y

**Published:** 2023-08-10

**Authors:** Wenrui Han, Yao Jiao, Sicong Mi, Shu Han, Junji Xu, Song Li, Yi Liu, Lijia Guo

**Affiliations:** 1 Department of Orthodontics, School of Stomatology, Capital Medical University, Tian Tan Xi Li No.4, Beijing, 100050 People’s Republic of China; 2 Laboratory of Tissue Regeneration and Immunology and Department of Periodontics, Beijing Key Laboratory of Tooth Regeneration and Function Reconstruction, School of Stomatology, Capital Medical University, Tian Tan Xi Li No.4, Beijing, 100050 People’s Republic of China

**Keywords:** Stevia, Periodontitis, Alveolar bone loss, *Porphyromonas gingivalis*, Mice

## Abstract

**Background:**

Excessive sugar intake has become a major challenge in modern societies. Stevioside is a promising non-calorie sweetener with anti-inflammatory effects; however, its effects on the oral environment and periodontitis remain unclear. Therefore, this study explores the effect of stevioside on periodontitis in mice.

**Methods:**

Mice were divided into four groups, namely, control, treated with water, and periodontitis models, established using 5 − 0 silk sutures ligation around the second molar then infected the oral cavity with *Porphyromonas gingivalis* (*P. gingivalis*) viscous suspension, divided into three groups treated with 0.1% stevioside (P + S), 10% glucose (P + G), or water (P). Micro-CT scanning was used to assess alveolar bone resorption, while RT-PCR was used to evaluate the inflammatory factors expression and *P. gingivalis* invasion in the gingiva. The composition of the oral bacteria was analysed using 16 S rRNA sequence in the saliva. In addition, *P. gingivalis* was co-cultured with stevioside at different concentrations in vitro, and bacterial activity was detected via optical density values and live/dead staining. The virulence was detected using RT-PCR, while biofilm formation was detected using scanning electron microscopy.

**Results:**

Compared with 10% glucose, treatment with 0.1% stevioside reduced alveolar bone absorption and osteoclasts while decreasing IL-6, TNF-α, IL-1β, and *P. gingivalis* in the gingiva of periodontitis mice. The CEJ-ABC distance in the P + S group was significantly lower than that in the P and P + G groups (*P* < 0.05). Moreover, the composition of the oral bacteria in the P + S group was similar to that of the control. In vitro stevioside treatment also reduced the bacterial activity and toxicity of *P. gingivalis* in a dose-dependent manner and affected its biofilm composition.

**Conclusion:**

Our results indicate that, compared with 10% glucose, 0.1% stevioside intake can reduce alveolar bone resorption and inflammation in periodontal tissues in mice; the bacterial composition following 0.1% stevioside intake was similar to that of a healthy environment. In vitro, high concentrations of stevioside reduced *P. gingivalis* activity, biofilm formation, and virulence expression. Therefore, stevioside is a potential alternative to glucose for patients with periodontitis.

**Supplementary Information:**

The online version contains supplementary material available at 10.1186/s12903-023-03229-y.

## Background

Sugar consumption has significantly increased [1], despite the World Health Organization recommending that daily free sugar intake should not exceed 25 g (approximately 5% of the energy ingestion). High intake of sugar is closely associated with obesity, cardiovascular disease [2], type 2 diabetes [3], chronic inflammatory diseases [4], periodontitis, and dental caries [5]. As such, low- and non-calorie sweeteners have become increasingly popular [6]; however, data on their effects are controversial, and different sweeteners have shown various biological characteristics.

The occurrence and development of periodontitis are closely associated with food, bacteria, and the immune response of the host. Previous studies have shown that high intake of sugar can increase alveolar bone resorption in rodent models of periodontitis [7]. In animal experiments, periodontitis could be observed after Lewis rats were fed with high sucrose and casein diet for 24 weeks without any mechanical damage [8]. Meanwhile, clinical studies have found that dietary intake of processed carbohydrates, such as sugar and trans fatty acids, promotes gingivitis and periodontitis, while consuming foods with low sugar and high micronutrients mitigates periodontitis and systemic inflammation [9–12]. Therefore, replacing caloric sugar (sucrose, fructose, glucose etc.) with non-calorie sweeteners may alleviate chronic inflammation and hyperglycaemia, thereby reducing the risk of periodontal disease.

*Stevia rebaudiana* is a plant from South American, nowadays the cultivation of it is rapidly increasing all over the world since it is being utilized as a natural non-clorie sugar substitute [13, 14]. Stevioside, one of the primary components of stevia, has anti-hyperglycaemic, anti-hypertensive, anti-caries, and anti-inflammatory properties [15–19]. In vitro, stevioside has been shown to inhibit the synthesis of inflammatory factors stimulated by lipopolysaccharide by inhibiting NF- κ B signal pathway and reducing the secretion of TNF- α through the TLR-4 pathway [19]. Stevia extract mouthwash can reduce plaque and gingival index in adolescents [20]; however, the effect of stevioside, and its underlying mechanism, on periodontitis remain nebulous. Therefore, this study investigates the effects of stevioside on periodontitis.

## Methods

### Experimental animals

Seven weeks old (body weight: 20–22 g) male healthy C57BL/6 mice were purchased from SPF Biotechnology Co., Ltd. (Beijing, China) and fed in the Laboratory Animal Center in Beijing Stomatological Hospital. Mice were acclimated to laboratory conditions for 7 days prior to experimentation. The study was conducted according to the guidelines of ARRIVE and the experimental protocols were approved by the Animal Care and Use Committee of the Beijing Stomatological Hospital, Capital Medical University, Beijing, China (KQYY-201,907–003).

### Induction of the periodontitis model

**Fig. 1:**
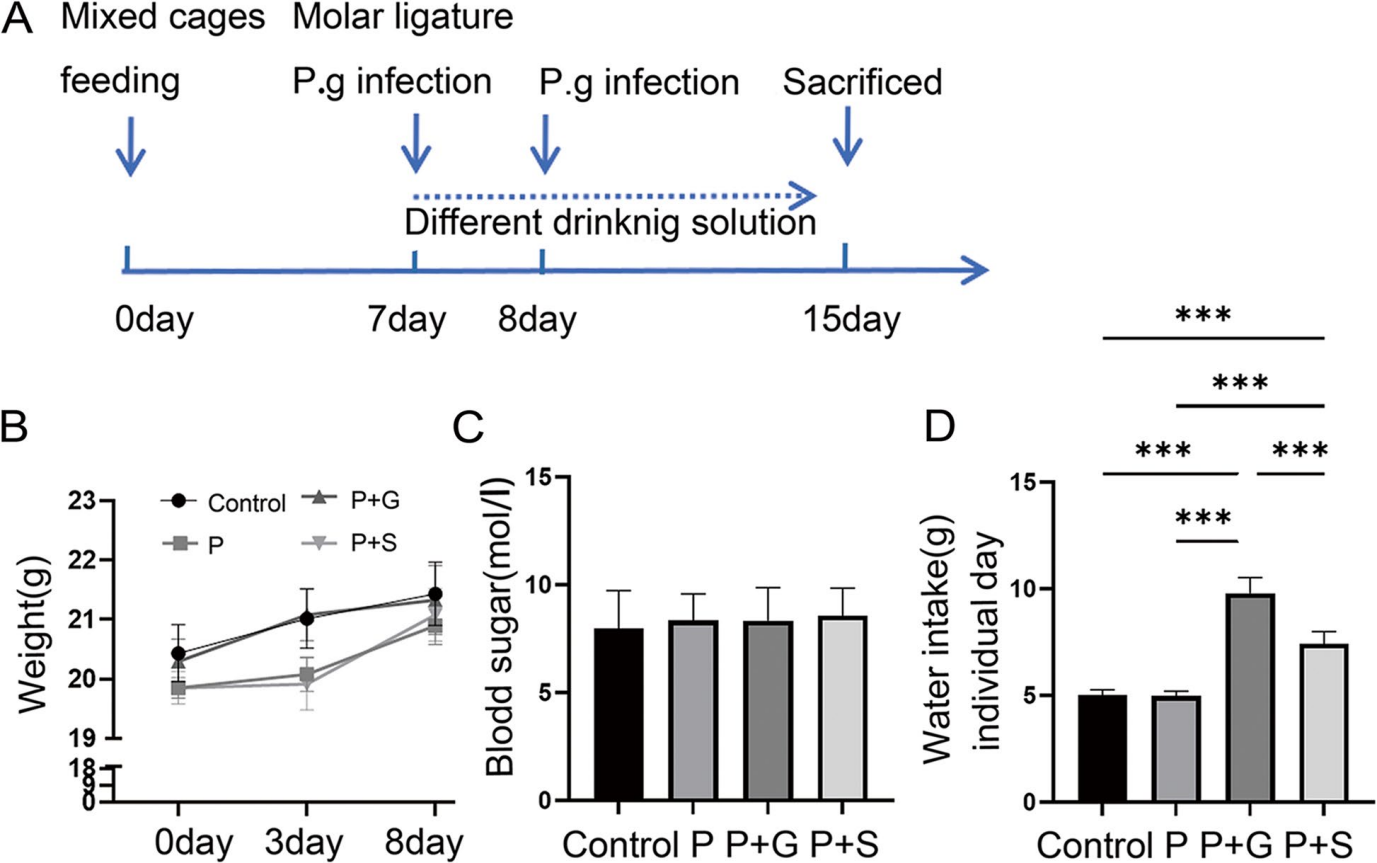
The general condition of mice after periodontal modeling. **A** A flowchart shows the time sequence in the in vivo experimental model. **B** Weights (g) of mice during 8 days of periodontal modeling. **C **Blood sugar (mol/l) of mice after 8 days of periodontal modeling. **D** Water intake (g) individual/day during periodontal modeling

A flowchart showing the time sequence in the experimental model is illustrated (Fig. 1A). A total of 24 mice were randomly divided into different groups: (1) Periodontitis + stevioside (P + S) group (*N* = 6), (2) periodontitis + glucose (P + G) group (*N* = 6), (3) the periodontitis (P) group (*N* = 6), and (4) the healthy (Control) group (*N* = 6). We referred to the commonly used quantities in most similar animal experiments and followed the 4R principles of animal experiments (reduction, refinement, replacement and responsibility) using the resource equation approach; the calculation formula is as follows: DF = N-k = kn-k, where DF is the degree of freedom, N is the total number of animals, k is the number of groups, and n is the number of each group; 10 ≤ DF ≤ 20 indicates that the sample size is appropriate [21]. The animals were anesthetized using pentobarbital sodium (40 mg/kg). According to the protocol for inducing periodontitis in mice [22, 23], 5 − 0 silk sutures (ARC Medical Supplies Co., Ltd., China) were used to perform ligation through the proximal and distal interdental gaps of the second molar. Subsequently, the sutures were looped around the second molar with forceps, making a triple-knot, and the excess sutures were cut with scissors.

Following ligation, sterilized cotton swabs were used to infect the oral cavity of mice with 10^9^ colony forming units (CFU) of *P. gingivalis* viscous suspension for 3 min. After modelling, mice were divided into four cages and given free access to the same soft food but different experimental drinking solutions for 8 days. Saliva was collected with sterilized cotton swabs for 1 min; mice were euthanized and fresh gingival tissue was taken for the follow-up experiments. Fixed the maxillae with 4% polyformaldehyde for 48 h, followed by micro-computed tomography (micro-CT) scanning, dehydration, and decalcification for sectioning.

### Exposure to different solutions and calculations of drinking water volume

After the periodontal model induction, the P + G mice were given 10% glucose (Servicebio, China) solution, the P + S mice were given 0.1% stevioside (Servicebio, China) solution, and water was given to the P and control groups. The concentrations were set based on previous studies and the sugar concentrations of commercially sold sweet drinks [24–26]. The solution in the drinking bottle was refilled daily, and the consumption amount was recorded and then calculated by the number of mice to estimate each animal’s daily drinking amount.

### Measurement of body weight and blood sugar in periodontitis

On the day of induction of the periodontitis model (day 7), animals first fasted for 4 h, then the tail vein blood was collected for blood sugar measurements with a blood glucose metre; the mice were also weighed to determine body weight. After 3 days, the mice were weighed again. On the final day of the experiment, the mice fasted for an additional 4 h, and tail vein blood was collected before sacrifice; the body weight was recorded for the third time.

### Measurement of alveolar bone resorption in periodontitis

**Fig. 2:**
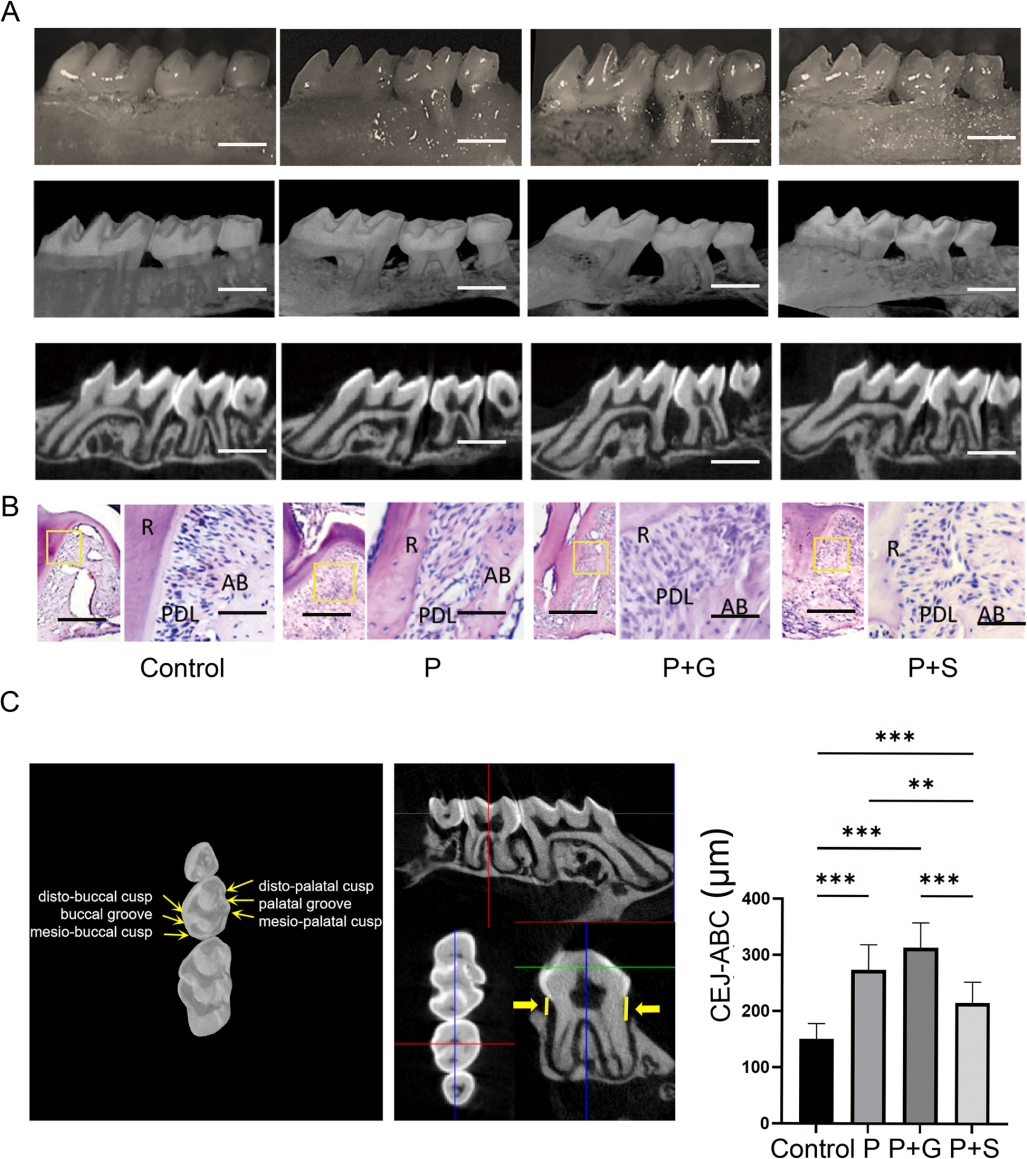
Stevioside exposure decreases the alveolar bone resorption of periodontitis in mice. **A** Stereoscopic microscope, micro-computed tomography scanning, and three-dimensional modeling views of alveolar bone resorption of periodontitis in different groups. **B** HE staining of periodontal ligament of the second molar in different groups. **C** The micro-computed tomography scanning shows that the CEJ-ABC distance (arrows) in the P+S group was lower than that in P and P+G groups (*P* < 0.05). *P < 0.05. **P < 0.01. ***P < 0.001. The scale bar in (A) was 1.0 mm. Scale bars in (B) were 500 μm and 50 μm

Alveolar bone resorption was assessed by the cemento-enamel junction to alveolar bone crest (CEJ-ABC) distance using an Olympus SZX12 Stereoscopic microscope (Olympus, Japan). Micro-CT (SkyScan, Belgium) scanning was used to analyse bone resorption and was scanned at an approximate thickness of 15 μm. We used Data Viewer to quantify the CEJ-ABC distance when the three molars were in the same direction and position by adjusting their roots so that all appeared in the image and were parallel (Fig. 2C), as previously described [27]. The same examiner performed the measurements three times and calculated an average value.

### Haematoxylin and eosin (HE) staining

Post micro-CT analysis, the samples were decalcified for 10 weeks using 10% EDTA, dehydrated using ethanol with different gradients, and embedded in paraffin; 5 μm-thick sections were cut along the mesial-distal direction and then treated with HE staining.

### Tartrate-resistant acid phosphatase (TRAP) staining and evaluation

Three sections from each animal were selected for TRAP staining to observe and evaluate osteoclasts in periodontal tissues. The staining was conducted according to the instructions of the Trap staining kit (Servicebio, China).

Osteoclasts were defined as TRAP-positive multinu-cleated cells containing three or more nuclei counted in three random areas in each section to calculate the number using brightfield microscopy.

### Cathepsin-K (CTSK) staining

We used CTSK staining to observe osteoclasts in periodontal tissues. After paraffin sections dewaxing, we used EDTA pH 9.0 solution (Servicebio, China) to repair the antigen then used 3% hydrogen peroxide solution (to avoid light incubation) for 25 min to block endogenous peroxidase, and finally, 3% bovine serum albumin for 30 min to block the serum. Subsequently, CTSK antibody (Servicebio, China) was added overnight at 4 ℃, followed by the goat anti-rabbit secondary antibody (Servicebio, China) at 25 ℃ for 50 min. Positive cells were treated with haematoxylin (brown colour) and observed under the microscope (Olympus, Japan).

### Measurement of inflammatory factors in mice gingival tissue

All gingival tissues were taken from the M1–M3 buccal side (from gingival to mucosal transition) and the palatal side (from gingival to 2 mm of the palatal) of mice. Total RNA was isolated using Trazol reagent (Cwbio, China); reverse transcription was completed and SYBR Premix Ex Taq^™^ was used to complete RT-PCR reactions according to the manufacturer instructions (Takara, China). The primers are listed in Table 1.

### **Measurement of** ***P. gingivalis*in mice gingival tissue**

All gingival tissues were collected as described above using a bacterial DNA extraction kit (Tiangen, China) and SYBR Green kit (Cwbio, China) to evaluate the relative content of *P. gingivalis* in the different groups using RT-PCR. The primers for universal bacteria 16s rRNA and *P. gingivalis* 16s rRNA are described in Table 1.

### Analyses of 16 S rRNA sequencing

To extract the DNA from the saliva sample, the V4 hypervariable regions of the 16 S rDNA were subjected to PCR amplification and library building. The library was quality analysed using Agilent Bioanalyzer 2100 and sequenced on the HiSeq platform. The representative OTU sequence was compared with sequences of the ribosome database project to identify the species. The raw reads were deposited in the NCBI Sequence Read Archive (SRA) database. (Accession Number: PRJNA937420).

### Bacterial strains and culture conditions

*P. g* ATCC 33,277 was cultured on blood agar mediums containing tryptic soy agar (Solarbio, China), supplemented with 1 µL/mL vitamin k3 (Landbridge Technology Co. Ltd., China) and 5 µL/mL hemin (Solarbio, China), in an anaerobic chamber under conditions of 85% N_2_, 10% H_2_, and 5% CO_2_ at 37 °C for 7 days. The growth curves were evaluated to establish the log phase under a 600 nm optical density (OD) to evaluate the CFU/mL concentration of microorganisms. *P. gingivalis* colonies were transferred to a 15 mL tube containing brain-heart infusion (BHI), hemin and menadione (Solarbio, China) and cultured for 48 h under anaerobic conditions.

### **Quantification of** ***P. gingivalis*co-cultured with stevioside in vitro**

The in vitro experiments were divided into five groups. The *P. gingivalis* culture was incubated in BHI, as previously described, until it reached an OD_600_ of 0.8 (about 1 × 10^9^ CFU/mL), then, transferred aliquots of 200 µL to a 6-well plate (Corning, USA). Different P + S groups were co-cultured in BHI in the presence of 5%, 1% or 0.1% of stevioside, and the P + G group was co-cultured in BHI in the presence of 10% glucose. The control group was cultured in BHI, and all bacterial solutions were filled to 2,000 µL with BHI and collected after 36 h. Three aliquots of 50 µL of solution were transferred from each well to a 96-well plate and the OD was measured under 600 nm using a plate reader (BioRad, USA). A total of 100µL of solution was transferred from each well to centrifugal tubes to make 10^− 1^ to 10^− 9^ dilution solution. Plates were labelled with 10^− 7^, 10^− 8^, and 10^− 9^ (3 plates per label) and 100 uL of the corresponding concentration solution was added to the plate and applied evenly with L-shaped spreaders (Solarbio, China). After 5–7 days of anaerobic culture, the number of colonies on each plate was counted (30–300 colonies were considered suitable).

### **The virulence factors of RNA expressions of** ***P. gingivalis*co-cultured with stevioside **in vitro

The in vitro experiments were divided into 4 groups based on the OD_600_ value. Different groups were co-cultured in BHI in the presence of 10% glucose or 1% or 0.1% stevioside. The control group was cultured in BHI, and all bacterial solutions were filled to 2,000 µL with BHI and collected after 36 h. The bacteria were collected in centrifuged tubes and centrifuged at 10,000 rpm for 1 min, following which a bacterial DNA extraction kit (Tiangen, China) and SYBR Green kit (Cwbio, China) were used to evaluate the relative expressions of Fim-A and Hag-A in different groups via RT-PCR. The primers of different genes are all listed in Table 1.

**Table 1:** Primers sequences of different genes in the RT-PCR

Primers	Sequence (5’ to 3’)
Gapdh F	TGCACCACCAACTGTTA
Gapdh R	GATGCAGGGATGATGTT
TNF-α F	CCACGTCGTAGCAAACCAC
TNF-α R	TTGTCCCTTGAAGAGAACCTG
IL-1β F	CAGGCAGGCAGTATCACTCA
IL-1β R	TGTCCTCATCCTGGAAGGTC
IL-6 F	CCGGAGAGGAGACTTCACAG
IL-6 R	TCCACGATTTCCCAGAGAAC
U-16 S rRNA F	ACTCCTACGGGAGGCAGCAGT
U-16 S rRNA R	ATTACCGCGGCTGCTGGC
Pg 16 S rRNA F	TGGGTTTAAAGGGTGCGTAG
Pg 16 S rRNA R	CAATCGGAGTTCCTCGTGAT
HagA-F	ACAGCATCAGCCGATATTCC
HagA-R	CGAATTCATTGCCACCTTCT
FimA-F	TACTTCCACGCCTTCTCCTGTT
FimA-R	CATCTTTACTGTTGCCACTTCG

### **Biofilm structure of** ***P. gingivalis*co-cultured with stevioside**

*P. gingivalis* was co-cultured on round glass coverslips (Solarbio, China) to allow for biofilm development for 36 h. The slices were fixed with 4% Glutaraldehyde (EM Grade) (Solarbio, China) for 2 h, and 30%, 50%, 75%, 85%, 95%, and 100% gradient ethanol for dehydration. After spraying gold on the slices, the morphology and structure of the biofilm were observed under a field emission scanning electron microscope (SEM) (Hitachi, Japan).

### **Live/dead staining of** ***P. gingivalis*co-cultured with stevioside**

The staining was performed using a live/dead BacLight Bacterial Viability Kit (Oregon, USA) after *P. gingivalis* biofilm development for 36 h, as described above. After repeated rinsing of the culture medium with saline, we mixed dye components A and B of equal volumes and added the mixture to the bacterial biofilm (3 uL/mL) and incubated at 25 ℃ in the dark for 15 min before observation with an Olympus BX61 microscope (Olympus, Japan). Live and dead bacteria were dyed green and red, respectively; corresponding images were merged.

### Statistical analysis

Statistics were analyzed by SPSS 32.0 software. Data are shown as mean ± standard deviation. Statistical significances were compared using Student’s *t-*test or one-way ANOVA analysis (*P* > 0.05, *P < 0.05, **P < 0.01).

## Results

### The general condition of mice showed different changes following periodontal modelling

The weight of mice in the control and the P + G groups increased rapidly, but there was no statistical difference after 3 days, while the weight of mice in the P and the P + S groups increased steadily and slowly; all weights tended to be consistent at 8 days (Fig. 1B). We recorded and estimated the average amount of drinking water for the different groups of mice daily and showed that the drinking amount of group P + G was the highest (*P* < 0.05) and that of group P + S was significantly higher than that in the remaining two groups (*P* < 0.05; Fig. 1C). After drinking different solutions for 8 days, no significant difference was found in blood sugar among the four groups (Fig. 1D).

### Stevioside exposure decreases alveolar bone resorption in periodontitis

To evaluate alveolar bone resorption in the four groups, we used a stereoscopic microscope, micro-CT scanning and three-dimensional modelling. The P, P + G, and P + S groups showed alveolar bone resorption in the inter-adjacent areas of the maxillary first molars, second molars, and third molars, indicating successful periodontitis modelling. Alveolar bone resorption in the P + G group was the most obvious, while in the P + S group was the least. To more accurately assess the alveolar bone resorption, the maxillary was scanned and modelled with CT Vox software. The results were consistent with body microscopy (Fig. 2A). HE staining of the periodontal ligament of the second molar showed that the P, P + G, and P + S groups had expended ligaments (Fig. 2B). Scanning of micro-CT showed that the CEJ-ABC distance in the P + S group was lower than that in the P and P + G groups (*P* < 0.05; Fig. 2C).

### **Stevioside exposure decreases the expression of inflammatory cytokines**, ***P. gingivalis***, **and the number of osteoclasts in periodontal tissue**

**Fig. 3:**
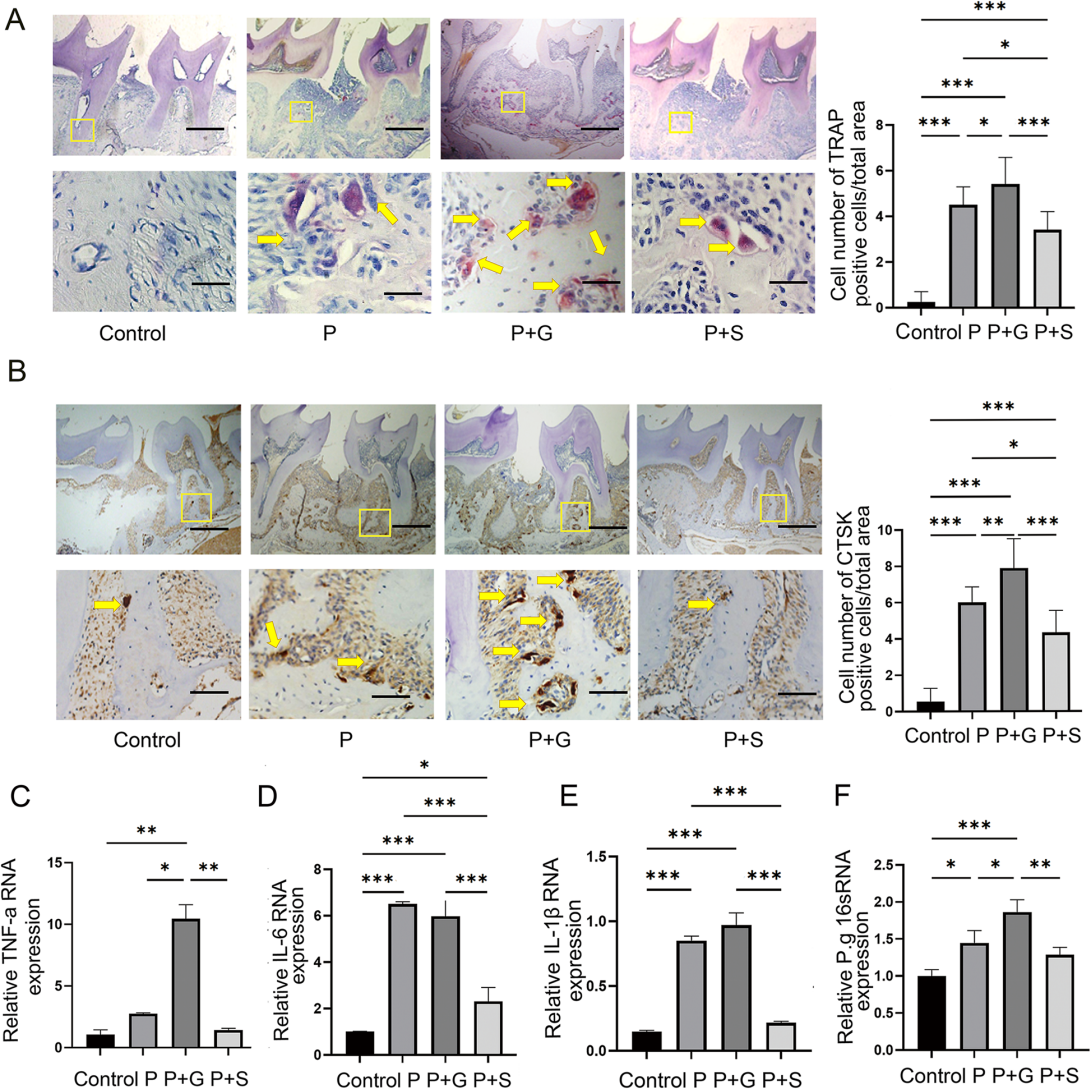
Stevioside exposure reduced the number of osteoclasts, the RNA expression of inflammatory cytokines, and P. gingivalis in periodontal tissue in mice. **A** Tartrate-resistant acid phosphatase (TRAP) staining (arrows). The osteoclast number of the P+S group was lower than that in P and P+G groups (*P*＜0.05). **B **CTSK staining (arrows) after modeling. The CTSK-positive number of cells in the P+S group was lower than that in P and P+G groups (*P* < 0.05). **C **The relative quantification of qPCR of TNF-a in gingiva tissues. **D **The relative quantification of qPCR of IL -1β in gingiva tissues. **E **The relative quantification of qPCR of IL-6 in gingiva tissues. **F **The relative quantification of qPCR of the 16S rRNA gene of *P. gingivalis* in gingiva tissues. *P < 0.05. **P < 0.01. ***P < 0.001. The scale bars in (A) and (B) were 500 μm and 50 μm

Upon histological examination, TRAP staining showed that the osteoclast number in the P, P + S, and P + G groups were significantly higher than that in the control. Among the three groups with periodontitis, the osteoclast number in the P + S group was the lowest, while that in the P + G group was the highest (*P* < 0.05; Fig. 3A). CTSK staining showed that the CTSK-positive cells number in the P, P + S, and P + G groups was significantly higher than that in the control. Among the three groups with periodontitis, the osteoclast number in the P + S was the lowest, while that in the P + G group was the highest (*P* < 0.05; Fig. 3B). The results of the two staining methods were consistent. We also investigated the expression levels of TNF-α in the P + G, IL-1β, and IL-6 in mice gingival tissue using PCR and demonstrated that TNF-α RNA expression was significantly increased in the P + G group compared with the other groups (*P* < 0.05; Fig. 3C). The expression of IL-β of the control group was the lowest, while that of the control and P + S groups was significantly lower compared with the P and P + G groups (*P* < 0.05; Fig. 3D). The expression of IL-6 in the control and P + S groups was significantly lower than that in the P group and P + G groups (*P* < 0.05; Fig. 3E). The results also demonstrated that the relative amount of *P. gingivalis* was the highest in the P + G group and second highest in the P group (*P* < 0.05; Fig. 3F).

### The 16 S rRNA results for oral bacterial changes after 8 days of periodontal modelling in mice

**Fig. 4:**
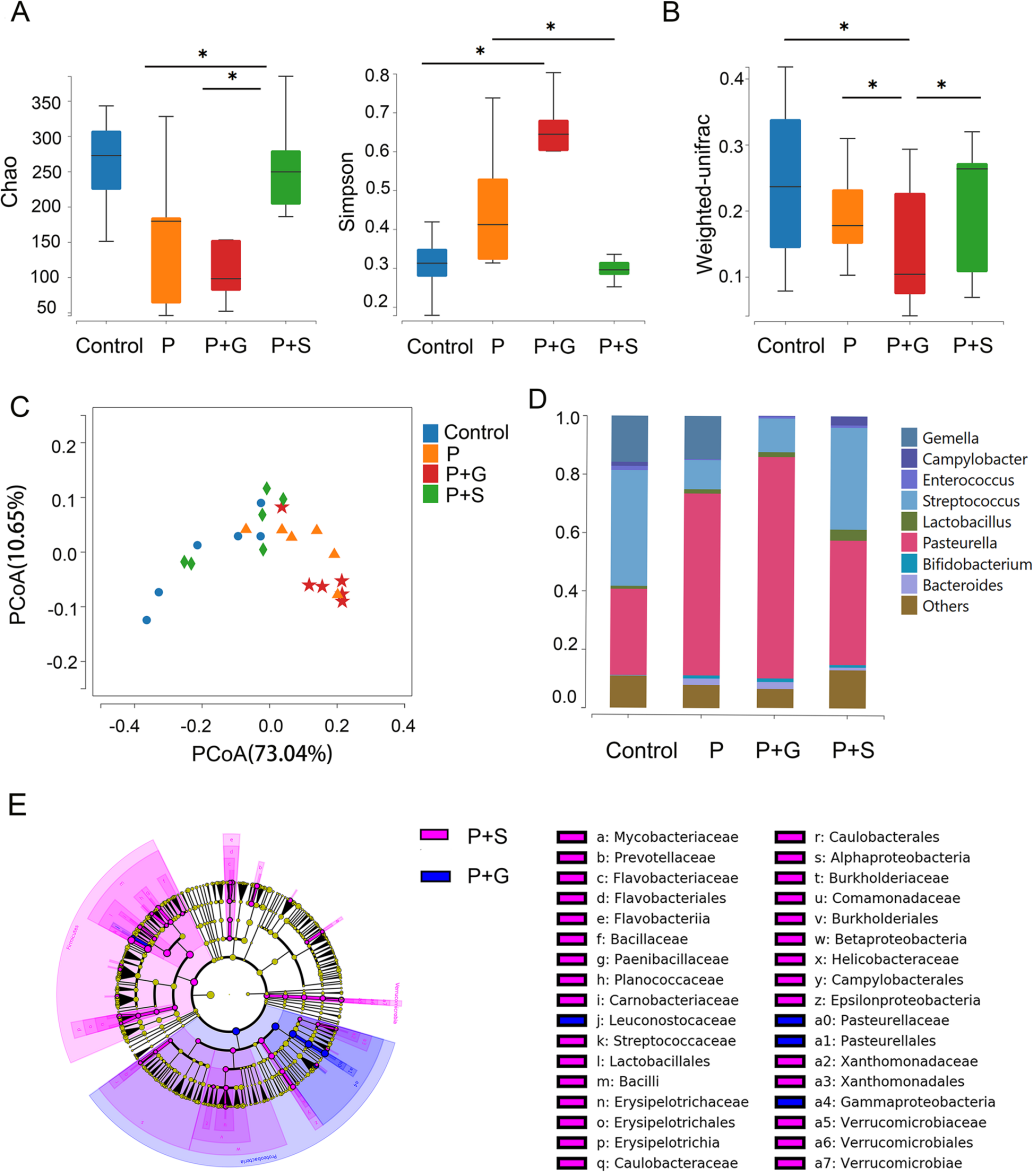
The 16 S rRNA results for oral bacterial changes after 8 days of periodontal modeling in mice. **A** The α-diversity (Chao and Simpson) of different groups after modeling. **B** The β-diversity of the different groups after modeling. **C** PCoA analysis showed the bacterial composition of different groups was separated. **D** Genus composition histogram of different groups after modeling. **E** LEfSe analysis showed the composition of bacteria of the P+S group and P+G groups vary from many levels. *P < 0.05. **P < 0.01. ***P < 0.001

The α-diversity (Chao) of the control and P + S groups after modelling was higher than that of the P and P + G groups, but only the P + S group showed a significant difference compared with the P and P + G groups (*P* < 0.05; Fig. 4A). The α-diversity (Simpson) of the control and P + S groups after modelling was lower than that of the P and P + G groups, and the control group showed significant difference compared with the P + G group; the P + S group showed significant difference compared with the P group (*P* < 0.05; Fig. 4A). The β-diversity of the P + G group after modelling was lower than that of the other groups (*P* < 0.05; Fig. 4B). The principal component analysis (PCoA) showed that the bacterial composition of the different groups was separated (Fig. 4C). Genus level histogram analysis showed that the composition of bacteria of the four groups was different. In the P and P + G groups, *Pasteurella* was the most common species, while *Streptococcus* tended to have a higher ratio in the control and P + S groups (Fig. 4D). LEfSe analysis also demonstrated that the bacteria of the P + S and P + G groups varied on many levels (Fig. 4E).

### **Stevioside reduces the growth and virulence of*P. gingivalis***

**Fig. 5:**
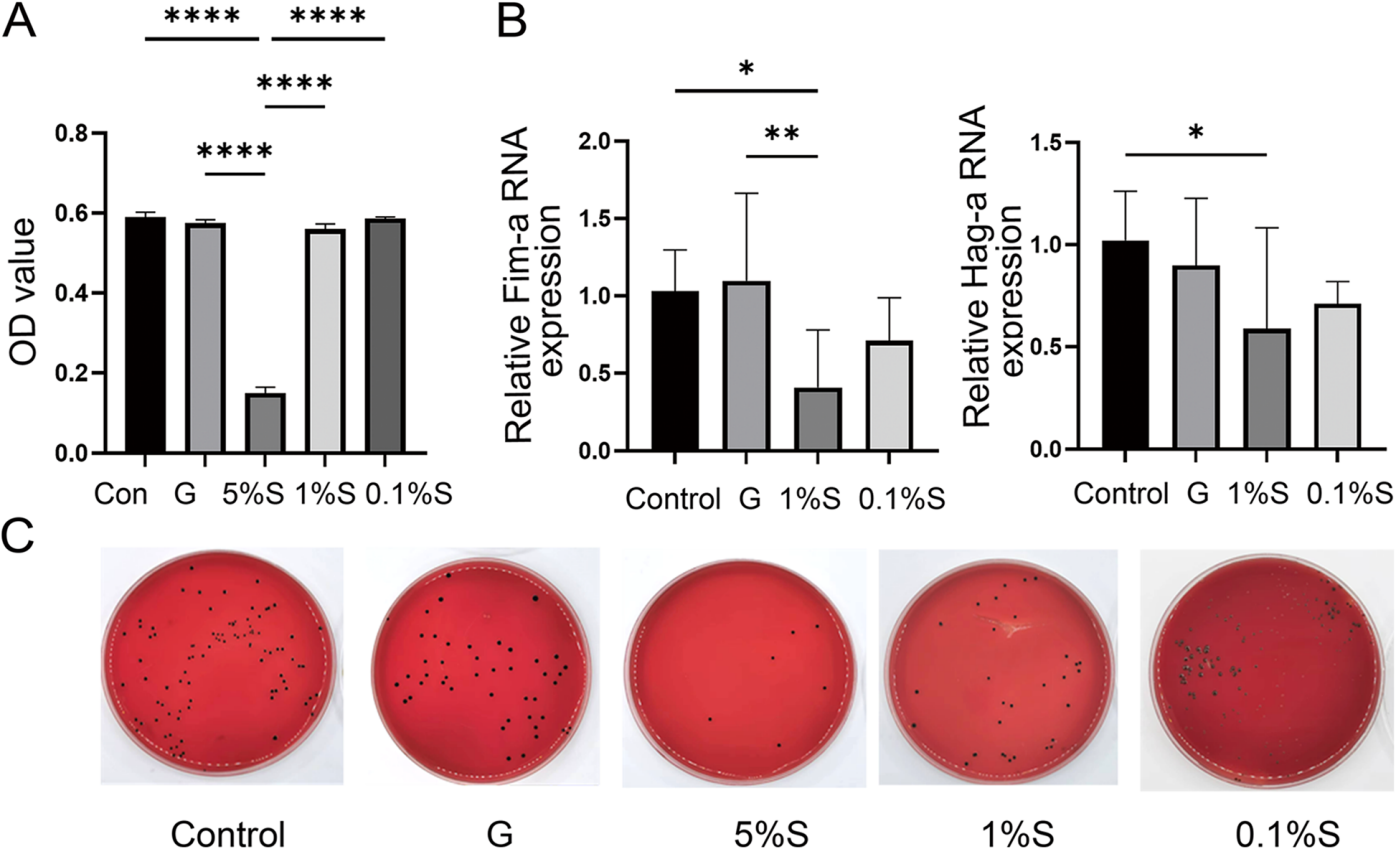
Effects of *P. gingivalis* co-cultured with different concentrations of stevioside on bacterial activity and virulence factors. **A** The OD value (600 nm) of *P. gingivalis* co-cultured with different concentrations of Stevioside. 5% stevioside significantly inhibited *P. gingivalis* growth (*P* < 0.05). **B** *P. gingivalis* co-cultured with different concentrations of stevioside,1% stevioside decreased the RNA expression of Fim-A and Hag-A (*P* < 0.05). **C** The number of active colony counts of *P. gingivalis* co-cultured with different concentrations of stevioside. *P < 0.05. **P < 0.01. ****P < 0.0001

After 36 h of *P. gingivalis* co-culture with different stevioside concentrations, the OD_600_ values of 10% glucose and 1% and 0.1% stevioside were not significantly different from those of the control group, while 5% stevioside significantly reduced the OD_600_ value of the bacterial solution (*P* < 0.05; Fig. 5A). We investigated the levels of Fim-α and Hag-α expression of *P. gingivalis* and found that Fim-α and Hag-α RNA expression decreased in the 1% stevioside group compared with that in the control group (*P* < 0.05; Fig. 5B). In the bacterial plate culture count experiment, the live colony number of 5% stevioside was reduced compared with that in the control, while 1% stevioside also showed a slight inhibition effect (Fig. 5C).

### Biofilm structure and live/dead staining

**Fig. 6:**
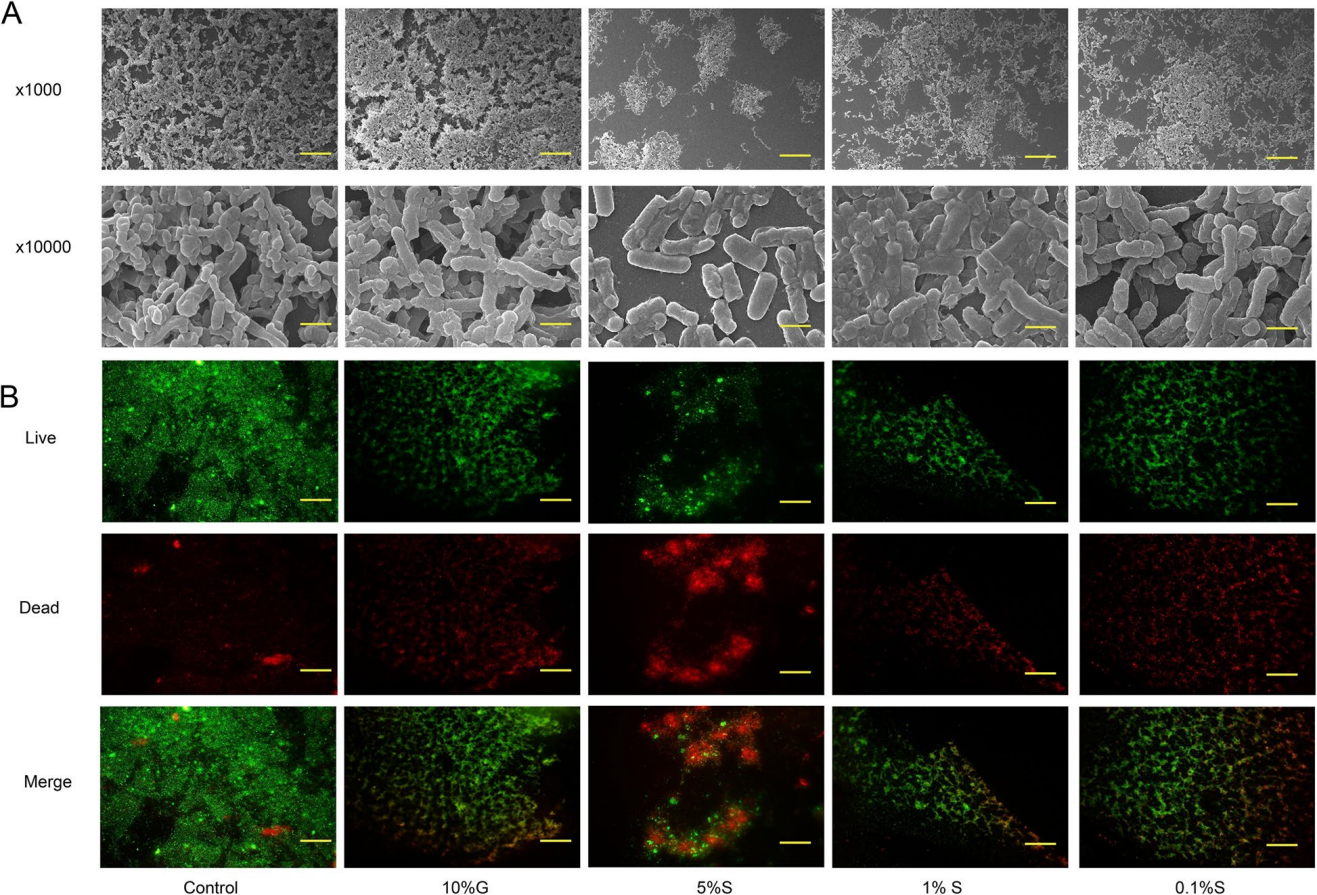
Biofilm structure and live/dead bacteria cells in the biofilm of *P. gingivalis* co-cultured with different concentrations of stevioside. **A** Biofilm structure of *P. gingivalis* in co-cultured with different concentrations of stevioside scanned by SEM. Magnification: x 1000 and x 10000. **B** Live/dead staining of *P. gingivalis* co-cultured with different concentrations of stevioside. Scale bars in (**A**) were 50 μm and 5 μm. Scale bars in (**B**) were 50 μm

After *P. gingivalis* and stevioside co-culture for 36 h, we observed that the control and P + G groups formed a dense multi-layer bacterial biofilm, while 5% stevioside significantly inhibited biofilm growth. As for 1% stevioside and 0.1% stevioside, the biofilm was established, but the number of layers and density decreased (Fig. 6A). In addition, the live/dead staining showed that the control group had few dead cells, while the 5% stevioside group had more dead cells than the other groups (Fig. 6B).

## Discussion

The main methods for inducing periodontitis in rodents are ligation and feeding periodontal pathogenic bacteria, among which the ligation method has a more obvious modeling effect, better site specificity, and high stability; therefore, it is the most commonly used and reliable periodontitis modeling method. Obvious periodontitis can be formed within 1 or 2 weeks [28, 29]. However, due to differences in manipulation, silk thread thickness and measurement sites, the value of the alveolar bone resorption differs; The alveolar bone resorption is between 150 and 250 μm [29, 30]. To better simulate the periodontitis environment with bacteria in a clinical setting, we combined two modeling methods, hence the alveolar bone loss was higher than that caused by ligation alone [31].

Our study found that alveolar bone absorption and inflammatory factors expression in the gingiva in the P + G group was the highest, consistent with previous findings in rats [32]. A high glucose environment can inhibit proliferation while promoting the apoptosis of periodontal ligament cells [33]. Therefore, in our study, the intake of 10% glucose could have caused a hyperglycaemic intracellular setting, which may have activated the electron transport chain to overproduce reactive oxygen species. There are four hypotheses to explain the possible connection between hyperglycaemia-induced reactive oxygen species and common complications: (1) It can increase the activity of the sorbitol pathway; (2) it can increase the formation of advanced glycation end product; (3) it can activate the protein kinase C isoforms; (4) it can increase the pathway flux of hexosamine [34–37].

In our study, however, the blood glucose levels in mice before and after the experiments showed no significant change. Similarly, one study found that a high glycaemic index intake increased gingival and periodontal inflammation, even when the level of blood sugar was excluded [38]. Previous studies have reported that high sugar and high-calorie intake have a negative inflammatory effect through early postprandial recurrent hyperglycaemia and increase the levels of late postprandial free fatty acids [38–40]. In the early stages, the hyperglycaemic cellular environment described above can occur, but detection is difficult. In the later stages, it seems that excessive consumption of carbohydrates promotes low-grade chronic inflammation through factors secreted by adipose tissue while increasing intestinal permeability and translocation of bacterial lipopolysaccharide into the portal vein; subsequently, inflammation in the liver is triggered via a mechanism dependent on toll-like receptor 4—a lipopolysaccharide receptor—as well as the presence of bacteria in the gut [41–43]. These factors are thought to lead certain species to produce excessive reactive oxygen and to release more pro-inflammatory cytokines, which may induce inflammation as well as periodontitis [44].

Stevia is a non-caloric sweetener with natural resources, of which stevioside is a primary component. Stevioside cannot be metabolized in the oral cavity and stomach and is eventually hydrolysed by intestinal bacteria into steviol [45–47]. In vitro and in vivo, stevioside showed anti-glycaemic effects by promoting the secretion of insulin, decreasing the concentration of glucose in plasma, and inhibiting the level of glucagon [7, 48]. Stevioside also has anti-viral effects, and anti-inflammatory effects by interfering with the NF-kappa B and mitogen-activated protein kinase signaling pathways, promoting the release of the anti-inflammatory cytokine, and inhibiting the pro-inflammatory protein and cytokines expressions [17–19, 49].

Considering the lack of significant differences in body weight and blood glucose among the groups and that stevioside is not directly metabolized by bacteria or cells in the oral cavity, which imply that the effects of stevioside and glucose on periodontitis may be more local than systematic. To further explore the effect of stevioside on the oral bacterial environment, we compared the oral bacterial microbiome of periodontitis mice.

The results of 16 S rRNA found that it changed after drinking different beverages for a week. α-diversity is “the average species diversity in a particular area or habitat”, while β-diversity is “the diversity of species between two habitats or the measure of similarity or dissimilarity of two regions” [50]. In our study, both α- and β-diversity analyses showed that the P + S and P + G groups had different characteristics. However, the β-diversity of the control and P + S groups were similar, while that of the P and P + G groups were similar. In other words, the species diversity, evenness, and uniformity of the control and P + S groups showed higher consistency.

We also found that both the P and P + G groups had more *Pasteurella*, which is a gram-negative bacterium that can infect mice, rats, guinea pigs, and hamsters, producing indole, urease, and hydrogen sulphide, which may lead to inflammation. It is a conditional pathogenic bacterium often mixed with other bacteria, and healthy animals can contain this bacterium in the respiratory and digestive tracts through trauma and reproductive organ infection. Animal disease occurs when their resistance decreases or if the virulence or number of bacteria is high [51]. Therefore, it is likely that stevioside influenced the composition and distribution of bacteria in the periodontal inflammatory environment in the oral cavity, making it more similar to the healthy oral bacterial environment in mice, although the underlying mechanism remains to be determined.

The P + G showed the most severe inflammation. Compared with the P + G group, alveolar bone resorption, osteoclasts and inflammatory factors were significantly decreased in the P + S group, suggesting that compared with drinking glucose beverage, using stevioside instead has a certain beneficial effect on reducing periodontitis. Compared with the P group, the amount of alveolar bone resorption, the number of osteoclasts and the number of IL-1 β and IL-6 in gingival in the P + S group were significantly lower. Besides, there was a significant difference in the bacterial composition between the two groups in the chao index. The two groups differed in the PcoA, and the difference was also observed on the lefse map at different levels (Supplementary Figure).

Since the subgingival fluid of mice is difficult to obtain, as a representative bacterium that can invade gingival tissue, the content of *P. gingivalis* can reflect the number of periodontal bacteria to some extent. Therefore, we detected *P. gingivalis* in the gingiva of mice and found that its content in the P + S group was the lowest. Therefore, we designed in vitro experiments to verify whether stevioside can affect periodontitis by affecting the activity or virulence of *P. gingivalis*.

In vitro, we found that 5% stevioside inhibited the growth of *P. gingivalis*, while 1% affected the virulence factor RNA expression of *P. gingivalis*. Unlike the in vivo experiment, 0.1% stevioside showed no obvious effect on the growth and virulence of *P. gingivalis*, which may be due to the in vivo interaction of a multi-bacterial environment. Another possible reason is that the in vivo experiment in mice lasted 7 days, while the in vitro experiment, limited by the logarithmic growth time of *P. gingivalis*, was only co-cultured for 36 h. *P. gingivalis* does not use sugar as the source of energy; therefore, a high concentration of glucose has little effect on its growth. However, why can stevioside inhibit the growth and virulence of *P. gingivalis*? There are two possible theories to explain this: One is that the abundance of terpenes—which can rupture cell membranes—is responsible for the antimicrobial effect of stevioside [52], while the other is that stevioside might lead to microbial imbalances, thereby disrupting the communication between gram-negative bacteria. It may also affect quorum sensing, which is an indispensable intra- and interbacterial communication system that regulates several bacterial community features [53].

The stevioside used for in vivo experiments in this study was 0.1% solution; the amount of stevioside added to daily drinks is generally within the rage of 0.05–0.5% [24].

We also considered the concentration of stevioside used in previous animal studies, such as the enteritis model [25], glucose tolerance metabolism model [30] and cariogenic model [26]. Furthermore, the sweetness of stevioside and consumption of sweet beverage in pre-experiment were also considered. The stevioside used for in vitro experiments in this study was 0.1%,1% and 5%. The purpose of choosing a high concentration in vitro is to determine whether the effects of stevioside on *P. gingivalis* are dose-depended. In terms of sweetness, 1% and 5% are not common concentrations used in the diet, but it may be used as a mouthwash or medicine in subsequent studies.

This study has some limitations. This modeling time was short, lasting only 8 days, which is insufficient to represent the relationship between long-term chronic periodontal disease and long-term sugar intake. To better observe the effects of different sugars on bacteria, long-term periodontal modelling remains warranted. Though animal experiments can better control for the mixed factors of diet and environment, most bacterial genera and species found in the oral cavity of mice do not exist in humans. In vitro research is limited in biological relevance, and oral microbial composition is a complex environment; therefore, further research is warranted to reveal the mechanism by which stevioside effects the microbial community in the human oral cavity.

## Conclusion

In this study, we observed that stevioside and glucose beverages have different effects on oral bacterial composition in mice. In vivo, compared with glucose, the effect of stevioside on bacterial composition was similar to that of the control, and reduced alveolar bone resorption and inflammation in periodontal tissues in mice.

Moreover, in vitro experiments showed that high stevioside concentrations could reduce *P. gingivalis* activity, biofilm formation, and virulence expression. Our results suggest stevioside maybe has the potential to be a beneficial alternative to glucose. However, clinical studies remain warranted to confirm the effects observed in this study on humans.

### Supplementary Information

**Additional file 1: Supplementary Figure. **LEfSe analysis of P+S group and P group. LEfSe analysis showed the composition of bacteria of the P+S group and P group vary from many levels.

## Data Availability

All data generated and analysed of this study are included in this published article and its supplementary information files. The 16 S rRNA datasets generated and/or analysed during the current study are available in the NCBI Sequence Read Archive (SRA), database (Accession Number: PRJNA937420, https://www.ncbi.nlm.nih.gov/sra/PRJNA937420 ).

## Abbreviations

ABC: Alveolar bone crest
BHI: Brain-heart infusion
CEJ: Cemento-enamel junction
CFU: Colony forming units
CTSK: Cathepsin-K
HE: Haematoxylin and eosin
Micro-CT: Micro-computed tomography
OD: Optical density
SEM: Scanning electron microscope
TRAP: Tartrate-resistant acid phosphatase
PCoA: Principal Co-ordinates analysis
