# Supplementary material for: Stevioside reduces inflammation in periodontitis by changing the oral bacterial composition and inhibiting P. gingivalis in mice
Source: BMC Oral Health. 2023 Aug 10;23:550. doi: 10.1186/s12903-023-03229-y (PMC10416424; doi:10.1186/s12903-023-03229-y)
Supplement: Supplementary file 1 — Additional file 1: Supplementary Figure. LEfSe analysis of P+S group and P group. LEfSe analysis showed the composition of bacteria of the P+S group and P group vary from many levels. [file 12903_2023_3229_MOESM1_ESM.docx]

**
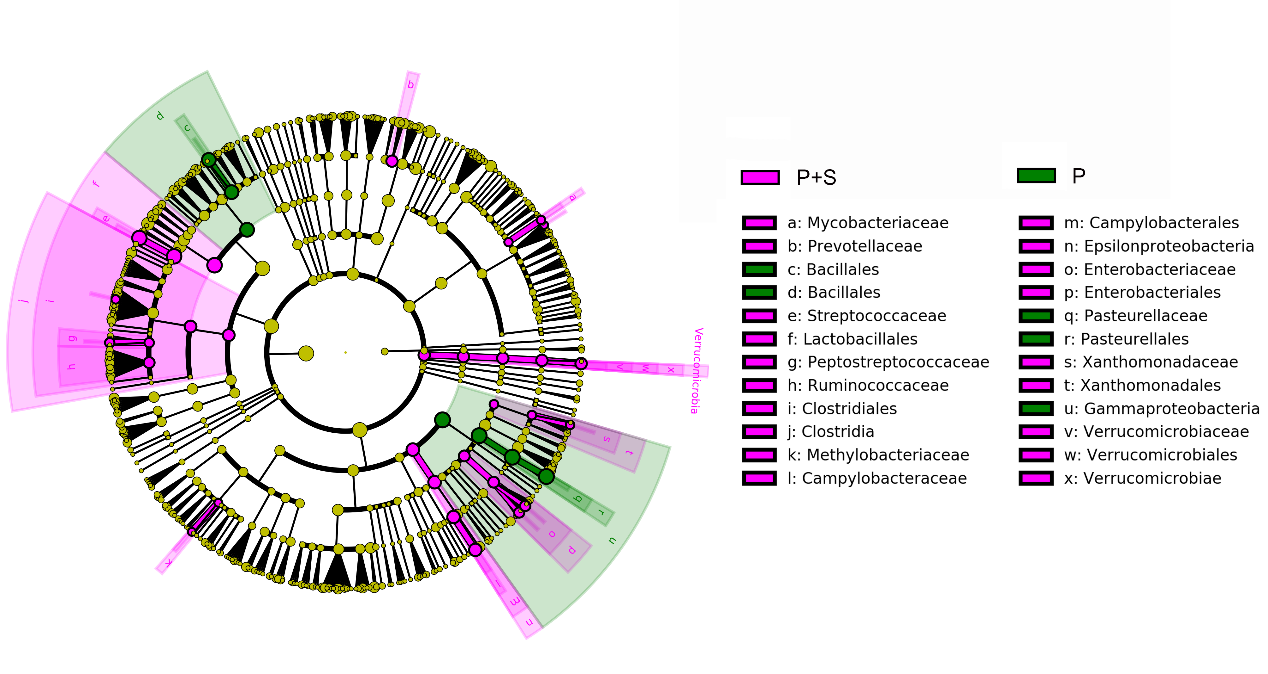
**

**Supplementary Figure. LEfSe analysis of P+S group and P group**

LEfSe analysis showed the composition of bacteria of the P+S group and P group vary from many levels.
